# The Equity of Health Care Spending in South Korea: Testing the Impact of Publicness

**DOI:** 10.3390/ijerph17051775

**Published:** 2020-03-09

**Authors:** Youngju Kang, Minyoung Kim, Kwangho Jung

**Affiliations:** 1 Korea Research Institute for Local Administration, Gangwondo 26464, Korea; kyj77@krila.re.kr; 2 Korea Institute of Criminology, Seoul 06764, Korea; milla.kim@gmail.com; 3 Korea Institute of Public Affairs, Institute of Information Knowledge and Policy, Graduate School of Public Administration, Seoul National University, Seoul 08826, Korea

**Keywords:** health care equity, sectoral difference, publicness, hospital ownership, nonprofit

## Abstract

This paper examined the important organizational and managerial factors of publicness for the equity of health care. The extent of organizational publicness was measured with key independent variables such as ownership, evaluation, and accreditation. The dependent variable was measured by three equity indicators for patients under medical care and veterans care: financial inequity, social equity, and overall equity. We analyzed unbalanced panel data with 328 general hospitals between 2008 and 2012. We performed panel analysis with fixed and random effects. Our findings illustrate that government ownership is significantly associated with differences in equity indicators. Government owned hospitals show the better performance for equity than nonprofit and individually owned hospitals do. Compared to nonprofit and individually owned hospitals, government owned hospitals have a higher share of medical payment bills and health care spending for the disadvantaged but a lower proportion of out-of-pocket payment. Government evaluation is also significantly related to better equity performance. There are, however, significantly negative interactions between hospital government ownership and the size of medical payment bills. We found a significant tendency that the more medical payments, the less responsiveness to the equity of health care in government owned hospitals. Future research in hospital performance is required to consider not only sectoral differences but also the negative proclivity of public hospitals that shrink health care services for the poor. Further research is also expected to explore what sectoral identities and behaviors across public, nonprofit, and private hospitals influence the level of equity or inequity in health care.

## 1. Introduction

In the last decades of the NPM (New Public Management) paradigm, economic performance indicators in the health care sector have been emphasized in the medical industry and hospital services. In this context, maintaining the equity of health services for the vulnerable is still an important challenge. Recently, the privatization of the health care in South Korea has become a controversial issue for health care service workers, civil organizations, and opposition parties. The increasing pressure of marketization causes hospitals to pay more attention to efficiency and effectiveness through adopting market-based policy instruments. However, recent research argues that privatization of health care would lower the quality of health care services, drive up health care costs, and deteriorate social performance of hospitals [1,2,3].

In general, people think that public organizations are not efficient as they do not try to seek profits because they have publicness characteristics such as non-distributional constraints, public goals, and political controls. Therefore, it is thought that they could serve the non-privileged persons better than private organizations. This is why people agree that the government should establish and support public organizations, especially in health care. From this perspective the fierce debate begins. However, the empirical results about organizational publicness and performance do not confirm what people have expected for the role of public organizations in healthcare. The results are mixed [1,2,3,4]. It is not clear whether public or nonprofit organizations would serve the less privileged people better than for-profit ones. Additionally, they mainly focused on economic performance or efficiency [5,6]. Considering the publicness of health care, equity is a most important part of the performance in health care among other criteria like quality, effectiveness, or efficiency, fairness, and access.

There are emerging concerns about the degree of equity in health care across three sectors in health care. However, few studies have used hospital level panel data to test social performance across different sectors of hospitals. Unlike previous studies, this research divides hospital ownership into public, nonprofit, and profit organizational status. While previous studies have not seriously distinguished between government and nonprofit sectors in the Western countries [3,7,8], government and nonprofit sectors are quite different in Asian countries. Nonprofit sector hospitals are more likely to pursue profitability and efficiency than government hospitals. Little empirical research has explored various differences between government agencies and nonprofit organizations in terms of organizational performance. Therefore, research on sectoral difference needs to distinguish government hospitals and nonprofit hospitals.

In order to accrue more evidence of a link between publicness and public service outcomes in health care, we tried to identify what the important organizational and managerial factors for the equity of hospitals in Korea are. More specifically, we explored the impact of publicness on social performance for the disadvantaged in Korean hospitals. We used three indicators of social performance in hospitals: (1) financial inequity measured by the ratio of out-of-pocket money to the total health care payment in hospitals, (2) social equity measured by the ratio of payment bills for the poor to the total payment bills in hospital, and (3) overall equity measured by the ratio of health care payment for the underprivileged to the total health care payment in hospitals. We used four elements about publicness in hospitals: ownership and external control variables such as government evaluation, government accreditation, and accounting audit. Derived from organization research and recent debates about publicness, our research suggests that public, nonprofit, and individually owned hospitals in South Korea are different from the level of social performance. We analyzed panel data on 328 general hospitals with a fixed effects model from 2008 to 2012.

## 2. Literature Review 

### 2.1. Expanded Theories on Publicness and Organizational Form

Little empirical research in the field of health care management has explored sectoral differences in diverse processes and outcomes of health care. Despite recent studies about the difference between public and nonpublic systems of health care [9,10,11,12], they have paid little attention to what characteristics are associated with the sectoral variances in health care outcomes. They have mainly focused on simple sectoral differences, rather than on specific sectoral variances of ownership, control, and evaluation. For instance, for-profit hospitals provide health care that is more expensive and lower quality than public hospitals. On the other hand, a lack of financial accountability allows nonprofit and government hospitals to be less efficient than for-profit hospitals [9]. Ownership status in health care organizations may involve different practices of cost management and control mechanisms in contracting out management [10]. In addition, hospital ownership may influence the type of clinical practice [11] and generate differences in health care like bariatric surgery [12]. More systematic review and analysis about specific sectoral differences in health care is required for a better understanding about how health equity varies from pubic and nonpublic hospitals.

In health care literature, for quite a long time, it was not common to consider the organizational publicness as an important factor for classifying hospitals. Historically, four variables have been used to classify hospitals: type of service, type of control, average length of stay, and number of inpatient beds [13]. However, ever since studies based on organizational publicness have emerged, organizations of health care services became an interesting sector to investigate, because they have so many types to investigate from purely public to purely private hospitals and also the middle ones [1,3,4]. Before closely examining these empirical studies on publicness and organizational performance in health care, we need to review the development of organizational publicness theories.

After reviewing a large body of research on comparing the performance of public and private organizations, Scott and Falcone [14] categorized the conceptual work of publicness in three main types: the generic approach, the core approach, and more recently, the dimensional approach.

In the generic approach, potential differences between public and private organizations are discounted. As they face similar constraints and challenges, main management functions, organizational processes, and managerial values are essentially the same across sector boundaries [15].

On the contrary, in the core approach, the fundamental differences between public and private organizations is emphasized with their ownership, the legal status: private firms are owned by entrepreneurs or shareholders and public organizations are owned collectively by members of political communities. Therefore, public organizations are under the influence of political forces, not market forces [16,17]. However, the core approach could divide organizations into only two discrete types like purely public or purely private organizations [17].

The most recent approach, the dimensional approach, explains that publicness is not a single discrete attribute like ownership. Bozeman [18] argues that no organization is wholly public or private and all organizations are public to some degree. Therefore, organizations could be lined up as more public on some dimensions and more private on other dimensions. This dimensional approach which emphasizes political authority or economic authority (markets) is widely accepted as an important approach for confirming differences in public or private organizations in their structure, functioning, and performance [19]. For instance, Bozeman and Bretschneider [20] identify and operationalize four dimensions of publicness: resource publicness, goal or agenda publicness, communications publicness, and the core dimension of ownership. Then, they examined US R&D organizations to solve the ‘publicness puzzle’ and concluded that both the core measure of ownership status and standards of other dimensions of publicness are complementary to identify essential effects of the organizational publicness.

### 2.2. Organizational Publicness and Market Competition: What Makes Better Performance?

There are three possible results for the relationship between publicness and organizational performance: (1) the equity of public organizations are better that of private organizations; (2) the equity of private organizations is better that of public organizations; and (3) there could be no difference between the equity of public organizations and that of private organizations [1]. How could we explain what characteristics of organizational publicness would bring better performance? There are some economic explanations why the performance is different by the organizational forms: non-distribution, market competition, and information asymmetry.

First, the nature of non-distribution matters to organization performance function. Unlike for-profit organizations, nonprofit organizations are barred from distributing their net earnings, even though they have gained a lot of surplus for their activities. This non-distribution constraint is the most distinct character of nonprofit organizations. On the contrary, the for-profit organizations have to raise profits and distribute them to investors every quarter or every year, they try to lower cost in many ways, and serve only capable persons. Consequently, they may pursue more cost-effective health care services and not the equity or quality of health care [21,22,23].

Second, market competition makes health care providers carefully respond to customers. If there are many service providers and some market competition exists, this will provide some incentives for all providers within the region to improve their quality. Typically, for-profit providers respond to competition more quickly and sensitively than nonprofit or public organizations in service quality in order to attract customers [21,22,23].

These explanations are based on the publicness theories that organizational performance is highly affected by ownership status, control mechanism, and organizational goals. That is to say, it demonstrates the importance and appropriateness of the dimensional approach in publicness theory.

Third, information asymmetry influences both consumers and providers in health care provision, especially patients’ preferences between public and private hospitals [22,23,24]. When the customers do not have enough information for the quality of services, it is hard to decide which provider to choose. Especially in healthcare services, searching for which hospital has highly qualified doctors or up-to-date medical devices is much harder. Even though the customers could get some information it is not easy to understand. This generates the information asymmetry between the customers and providers, which may bring the opportunistic actions of for-profit organizations [24]. Therefore, people have some tendency to choose public organizations, believing the public or nonprofit organizations would serve them under the voluntary public service motivations.

### 2.3. Comparing the Equity of Public and Private Healthcare Organizations

The performance of health care could be evaluated with many criteria like quality (satisfaction), effectiveness or efficiency (profitability), fairness, and access. Among them, when we consider the publicness, equity is most important criteria in the health sector. Equity means that relevantly similar cases must be treated in similar manners and that relevantly different cases must be treated in different manners. Equity could be divided in two concepts, horizontal equity and vertical equity. Horizontal equity allocates equal or equivalent resources for equal needs; vertical equity allocates the allocation of different resources for different levels of need. For the policy implication, vertical equity gets more attention from policy makers, because it can be considered positive discrimination [25,26]. As targeted programs for the poor would redistribute resources form the rich to the poor, these would often face greater political obstacles. However, when considering the increasing gap in health among different socioeconomic groups, vertical equity is what the public organizations need to pursue and the government should control.

However, the empirical results do not confirm whether vertical equity could be achieved in health care, especially in public or nonprofit hospitals. The results are not conclusive.

After a systematic review on the four performance criteria (access, quality, cost/efficiency, and/or amount of charity care) of 149 peer-reviewed articles, Roseau and Linder [1] found for-profit superiority, nonprofit superiority, or no difference/mixed results. Overall, the nonprofits were judged superior 59% of the time, the for-profits superior only 12% of the time, and for the rest (29%), no difference was found or results were mixed.

Many studies support that public or nonprofit are better regarding equity. Norton and Staiger [27] focused on horizontal equity of hospitals by examining the effect of hospital ownership on the delivery of service to uninsured patients. The results show that when for-profit and nonprofit hospitals are located in the same area, both of them serve an equivalent number of uninsured patients, but for-profit hospitals tried to avoid the uninsured more often than nonprofit hospitals. Trivedi and Grebla [28] found that for persons aged 65 years or older, the Veterans Affairs health-care system significantly outperformed private-sector Medicare Advantage plans and delivered care that was less variable by site, geographic region, and socioeconomic status. Additionally, Barsanti and Nuti [29] found that the health care performance evaluation system addresses not only health care inequalities, but also confirms that the health system responds appropriately to different socioeconomic groups.

However, some suggest that there is little or no difference between that of public or nonprofit and for-profit hospitals on equity. Several studies show that for-profits may serve relatively high levels of charity care because they could charge costs more than nonprofits [1,28].

## 3. Research Frame and Method

This section may be divided by subheadings. It should provide a concise and precise description of the experimental results, their interpretation, as well as the experimental conclusions that can be drawn.

### 3.1. Comparing Organizational Form and Equity in Health Care Services

As Bozeman and Bretschneider [20] suggested, we encompassed four dimensions of publicness in order to more effectively assess the effects of an organization’s internal structure and external influences on performance. Primarily, we focused on the equity dimension among various health care performance indicators, such as quality, efficiency, equity, fairness, and access. The reason is that delivering to appropriate health care citizens who do not have enough resources for health care is one of the basic reasons why the government supports public hospitals with its administrative and financial resources. For this purpose, we analyzed how the dimensions of publicness such as ownership public control, and governance affect the social and financial equity after controlling for organization size and age (see Table 1).

### 3.2. Variables and Data

This study used panel data set with 339 tertiary hospitals from 2008 to 2012. The tertiary hospitals are regularly reviewed by the Health Insurance Review and Assessment Service for the medical cost and healthcare quality assessment services under the National Health Insurance Act. The data for our test were drawn from a wide range of sources. Appendix A and Appendix B provide a detailed description of the variables and their sources.

South Korea has three parts of social security including (1) four social insurances for pension, health, employment, and industrial accident compensation, (2) public assistance, and (3) social welfare services for the disabled, the aged, and women and children. This paper focuses on how hospitals can effectively contribute to promoting a public assistance program through providing health care for the disadvantaged. The public assistance program provides four programs for the disadvantaged such as a national basic livelihood security system, medical aid for the poor, veterans relief, and disaster relief.

Dependent variables are three equity measures which come from two public assistance programs for the disadvantaged groups from the poor to the veterans in medical care. We assessed, for each hospital, the equity of health care by three parts: financial inequity, social equity, and overall equity. Financial inequity was measured by the percentage of out-of-pocket payments in all the medical reimbursements for each hospital. Out-of-pocket spending included deductibles, coinsurance, and copayments for covered services plus all costs for services that are not covered by the national health insurance program. An increase of out-of-pocket proportion in the total revenue of hospital implies that the poor are likely to pay more for medical care from their own pockets. Social equity was assessed by the percentage of patients under the medical aid program and veterans care relief in all the medical payment statements for each hospital. Overall equity was measured by the percentage of the payment for the medical aid program and veteran relief program in all the medical payments.

Publicness variables included various organizational characteristics. First, ownership was measured in terms of public, nonprofit, and private status of hospitals. The value of the dummy variable of ‘Govt’ was 1 if the hospital is a national hospital or provincial or city hospitals owned by local governments. Otherwise, the value was zero. The others include the nonprofit and private hospitals. The nonprofit hospitals are owned by foundation corporation or social welfare foundation or educational foundation or medical corporation. The private hospitals are privately owned. Second, external control for publicness involved various public interventions from evaluation by the government, to healthcare accreditation, and to accounting audit.

Control variables included the total number of medical payments, the number of hospital beds, and hospital age. The number of payments and hospital beds represent organizational size. Normally, organization size is controlled because of its economics of scale which increases efficiency. However, we controlled these variables because organizations with many hospital beds and payments have more capacity to serve disadvantaged patients effectively and they tend to have more access to resources and ability to buffer influences of the organizational environment.

### 3.3. Analytical Methods

We used an unbalanced panel data containing 339 hospitals for the period 2008–2012. The unbalanced panel consists of 338 hospitals with different periods, excluding one hospital with missing values. Appendix C shows specific information about the coverage of panel data over time. While panel data can involve unobserved heterogeneity correlated with the independent variables, the fixed-effects model can control for time-invariant differences between the hospitals, so the estimated coefficients of the fixed-effects model are free from omitted time-invariant characteristics in hospitals. The fixed effects model can avoid heterogeneity bias derived from different hospitals. In contrast, the fixed effects approach can neglect a large amount of information about the potential effects of theoretically critical factors that do not change over time [29]. The typical fixed effects model ignores the ‘between effects’ from rarely time-invariant variables such as ownership characteristics of hospitals [30]. It is, however, necessary to consider the time-invariant factors if they contain crucial research questions. Our research attempts to explore the effects of publicness (i.e., ownership dummy variables) that come from higher level entities beyond individual hospitals. For the fixed effects model, we can partially identify the effects of hospital ownership by not only using an unbalanced panel data format and but also introducing interaction terms with time variant factors like the number of medical payments. On the other hand, we conducted the random effects model, but their estimates were not consistent. Relying on the Hausman test, we attempted to identify a potential correlation between the regressors and the individual hospital-specific random effects that makes regression coefficients in the random effects model inconsistent. Our Hausman tests favored the fixed effects model over the random effects approach.

The Stata statistical package ((Release 13, College Station, Texas, USA: StataCorp LP) was used to perform an unbalanced panel analysis. We used the following unbalanced panel regression model to estimate the effect of publicness on health care equity with interaction terms between the publicness elements and the number of medical payments (see Equations (1) and (2) below).

(1) $$y_{it}=\alpha +\beta_{j}Publicness_{itj}+\gamma_{k}Z_{itk}+\mu_{i}+\varepsilon_{it}$$

—*y_it_* represents the degree of equity performance where *i* = hospital and *t* = time

(*y_1_* = Financial inequity; *y_2_* = Social equity; *y_3_* = Overall equity)

—*Publciness_itj_* represents the number of *j* variables related to publicness as higher-level entities beyond individual hospitals

—*β_j_* is the coefficient for the publicness variables such as ownership, government evaluation, and accounting audit 

—*Z_itk_* is the number of *k* control variables

—*y_k_* is the coefficient for *k* control variables

—*u*_i_ (*i* = 1….n) is the intercept for each entity (n hospital-specific intercepts)

–ε*_it_* is the error term

The group of publicness variables include hospital ownership, government evaluation, hospital accreditation, and accounting audit. For instance, if the effect of a time-varying medical payment growth is different for public and nonpublic hospitals, the researcher needs to know this. Such relationships could be in opposite directions for different types of higher-level entities. The effect of payment increase on equity is negative in public hospitals compared to nonprofit hospitals.

The group of control variables representing Z include the number of payment statements, the number of hospital beds, and hospital age. In addition, the model considered interaction terms between hospital ownership and the number of payment statements. This panel data model allows us to estimate the effects of the publicness factors on social performance of hospitals after controlling for the unobserved elements over time representing the fixed characteristics of hospitals.

The details on this panel regression model is as follows in Equation (2).

(2) $$\begin{array}{ll} y_{it}=\alpha +\beta_{1}Govt_{it} & +\beta_{2}Govt\_EV{}_{it}+\beta_{3}Credit_{it}+\beta_{4}Audit_{it}\\ & +\beta_{5}Govt_{it}\times Ln\_Pay\_Count{}_{it}+\beta_{6}Private_{it}\\ & +\beta_{7}Private_{it}\times Ln\_Pay\_Count{}_{it}+\gamma_{1}Ln\_Pay\_Count_{it}+\gamma_{2}Age_{it}\\ & +\gamma_{3}Bed_{it}+\gamma_{4}\mathrm{Year}+\mu_{i}+\epsilon_{it} \end{array}$$

*Dependednt variables*: *y_1_* = The percentage of out-of-pocket payments of the medical expenses for those with medical aid (Financial inequity)

*y_2_* = The percentage of the number of medical payment statements for patients under medical care and veterans care (Social equity) 

*y_3_* = The percentage of medical aid and veteran payments in total hospital medical payment (Overall equity)

*Independent variables*: *Govt* is a dummy variable representing government ownership of a hospital (i.e., public hospital).

*Govt_EV* is a dummy variable representing whether or not hospitals are evaluated by the Ministry of Health and Welfare.

*Credit* is a dummy variable representing whether nor not hospitals received an accreditation granted by the Korea Institute for Healthcare Accreditation.

*Audit* is a dummy variable representing whether or not hospitals are monitored by external reviewers in terms of compliance audit, financial audit, and performance audit.

*Private* is a dummy variable representing the private ownership of a hospital.

*Ln_Pay_Count* is a total number of payment statements for each hospital transformed by natural logarithm.

*Int_govt* is an interaction term between *Ln_Pay_Count* and *Govt*

*Int_private* is an interaction term between *Ln_Pay_Count* and *Private*

*Bed* is the number of beds for each hospital

*Age* is the foundation year representing organizational age 

*Year* is the year dummy variable representing year specific fixed effects in panel data 

*i = 1, 2, 3, … N (N = 338)*

*t = 2008, 2009, 2010, 2011 2012 (T = 5)*

## 4. Empirical Findings and Discussions

### 4.1. Trends of Equitable Performance in Hospitals

Overall the financial inequity decreased while social equity and overall equity did not increase from 2008 to 2012 (see Table 2). This trend may be different from the degree of publicness of hospitals. The level of equity is different from whether or not hospitals have public ownership and are under government evaluation (see Table 3). Financial inequity is lower in public hospitals and also lower in hospitals under government evaluation, while social equity and overall equity are higher in public hospitals and hospitals under government evaluation (see Table 4). These differences in three types of equities are still clear between public and nonpublic hospitals (see Table 5) and between hospitals with and without government evaluation (see Table 6) during the period between 2008 and 2012.

**Figure 1:**
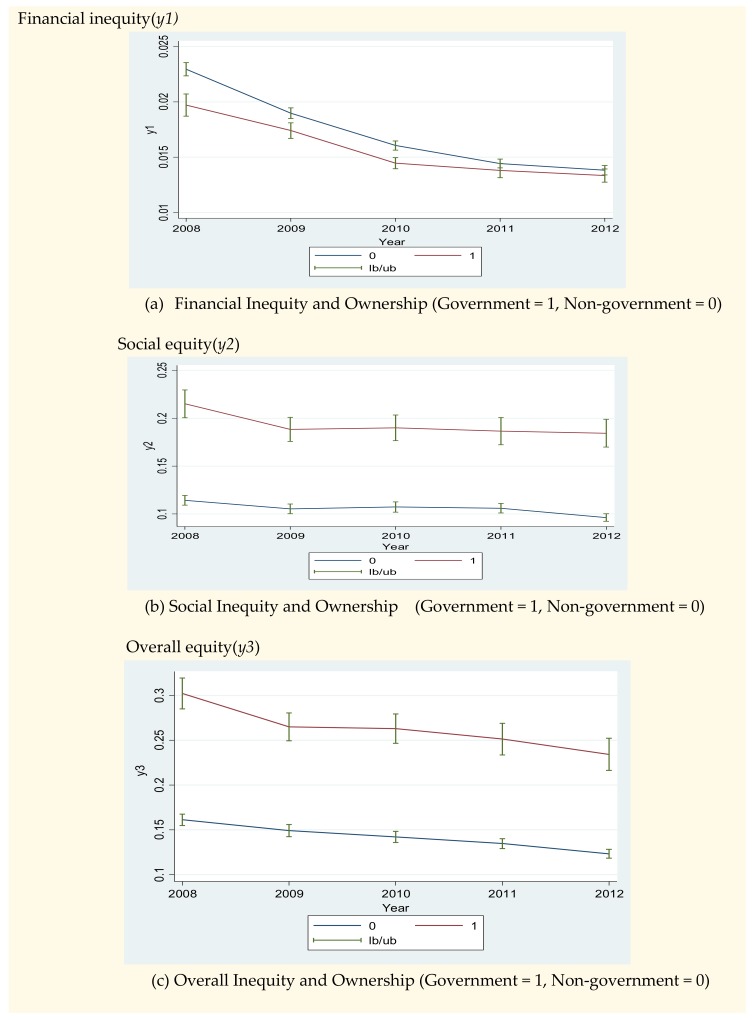
Comparing equity Indicators between public and nonpublic hospitals (2008–2012). Note: lb and ub are lower and upper boundaries of the 95% confidence level.

Figure 1 shows that financial inequity diminished from 2008 to 2012 (see Figure 1a) but social equity and overall equity did not increase and even decreased during this period (see Figure 1b,c). The decrease of financial inequity implies that the proportion of out-of-pocket payments in the total hospital payments has reduced and that the coverage of national health insurance and medical care assistance has expanded in the total payments in hospitals. On the other hand, the decrease of social equity implies that the proportion of medical payments for the poor with the Medical Care Assistance (MCA) program decreased. However, it should be noted that the total medical payments for the poor did not decrease, while the total medical payments for the patients with National Health Insurance (NHI) program increased (see Appendix D). The ratio of the number of medical payments with MCA to that with NHI would be decreased, which resulted in the decrease of the indicator of social equity. This case is the same as the case of the indicator of overall equity.

**Figure 2:**
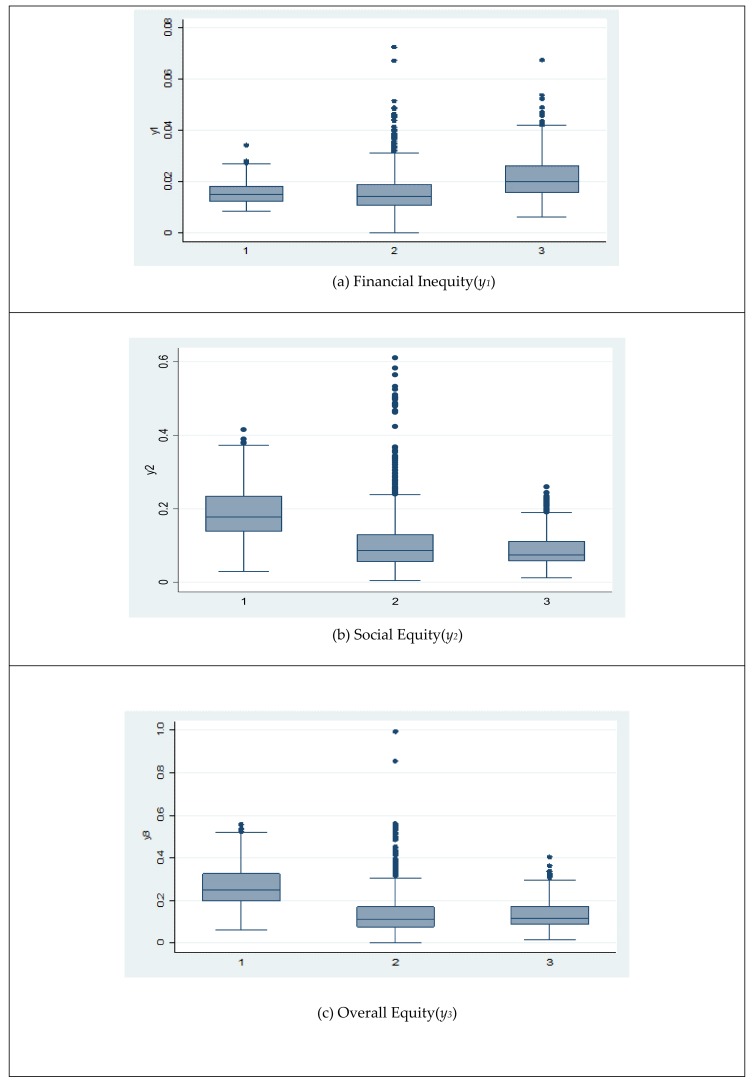
Comparing equity indicators across different sectors (2008–2012). Note: 1 = Government ownership, 2 = nonprofit organization ownership, 3 = individual ownership

Figure 2 shows differences in the variations of three equity indicators across three sectors. The proportion of out-of-pocket payments appears to be larger in individually owned hospitals. Both social equity and overall equity are higher in public hospitals directly owned by central and local governments, while these two equity indicators are similar in nonprofit hospitals and individually owned hospitals.

### 4.2. The Impact of Publicness on Social Performance in Hospitals

Table 7 shows how the variables about publicness are associated with financial inequity measured by the proportion of out-of-pocket payments. For the variables of publicness, public ownership of hospitals has a negative effect on financial inequity. In other words, the proportion of out-of-pocket payments in public hospitals is lower than that in nonprofit hospitals. The proportion of out-of-pocket payment decreases by 3.93% in public hospitals compared to nonprofit hospitals. The proportion of out-of-pocket payment also reduces by 2.36% in individually owned general hospitals compared to nonprofit hospitals. However, as the number of payment statements increases, both public and private ownership increases the proportion of out-of-pocket payments. This implies that an increase in the number of medical payment statements increases the proportion of out-of-pocket payments, where the proportion of out-of-pocket payments is still lower in the public and private hospitals than in nonprofit hospitals. It appears that both public and private hospitals attempt to maximize the revenue of health care spending through reducing the proportion of out-of-pocket payments as the number of medical payments increases. In addition, the proportion of out-of-pocket payments is lower in general hospitals under government evaluation than those not under government evaluation. It appears that government evaluation makes general hospitals consider the reduction of out-of-pocket payments through emphasizing public support of medical care in public health insurance and medical care assistance for the poor. The accreditation of hospitals does not have any impact on the reduction of the proportion of out-of-pocket payments. On other hand, the variable of accounting audit increases the proportion of out-of-pocket payments by 0.44.

An increase in the number of medical payment bills (Ln_Pay_Count) is likely to reduce the proportion of out-of-pocket payments. The number of medical payment bills consists of two parts: one from the public insurance program and medical insurance program (A) and the other from out-of-pocket money (B). As the total number of medical bills increases, the number of medical payments covered health insurance and medical assistance programs (A) increases, but the number of out-of-pocket payment bills (B) are relatively constant. As a result, an increase of the number of medical payment bills is likely to decrease the proportion of out-of-pocket payments. There are, however, strong significant interactions between government ownership and the size of the medical payment. The coefficient of the interaction term between public hospital dummy and the number of medical payments is statistically significant (0.0047, *p* = 0.007). The interaction coefficient between private hospital dummy and the medical payment variable is also significant (0.0028, *p* = 0.001). The interaction term implies that for public hospitals, a 10% increase in medical payment may result in a 4.7% increase in financial inequity, and for individual owned hospitals, a 2.8% increase in financial inequity, compared to nonprofit hospitals. In addition, both hospital age and the number of hospital beds are not related to the proportion of out-of-pocket payments.

Table 8 shows what factors in general hospitals are related to social inequity. Public ownership variables increase social equity when compared to nonprofit hospitals. On the other hand, in the case of the public ownership variable, the increase in medical payments of general hospitals has a negative effect on social equity. The interaction term between the number of medical payments and government owned hospitals (Int_govt) involves a negative coefficient (−0.0607, *p* = 0.001). In other words, even as public hospitals grow in size, their commitment to social equity may decrease. Private ownership, on the other hand, did not affect social equity. In general hospitals that receive government evaluation, social equity was about 15% higher than that of nonprofit hospital hospitals. Even in the case of government-accredited general hospitals, there was no significant difference in social equity compared to general hospitals without accreditation. On the other hand, social equity was lower in general hospitals under accounting audit than that of general hospitals without an audit monitoring system. An accounting audit system in general hospitals is not related to social equity. It appears that the audit mechanism diminishes the scope of social equity.

Table 9 shows what types of publicness are related to overall equity in general hospitals. Basically, both public ownership and government evaluation factors significantly increase the degree of overall equity. This implies that public hospitals are much more likely to increase the proportion of medical payments for the disadvantaged than nonprofit hospitals. The degree of overall equity in public hospitals is much larger by 84.5% compared with nonprofit hospitals. In other words, public ownership factor does really matter to the variation of overall equity in general hospitals of South Korea. However, an increase of medical payments in public hospitals tends to decrease the overall equity. The negative interaction term between the number of medical payments and government owned hospitals (Int_govt) is highly significant (coefficient = −0.0943, *p* < 0.001). The government evaluation factor contains a positive signal to promote overall equity. On the other hand, government accreditation and accounting audit are not related to overall equity.

## 5. Conclusions

In this paper, we made an initial empirical assessment of the relationship between the publicness and the equity of health care with a panel data set with 328 general hospitals from 2008 to 2012. In summary, the ownership of hospitals has the greatest impact on hospital social performance. First of all, public hospitals have lower levels of financial inequity than nonprofit and private hospitals. Public hospitals also have higher social equity and overall equity than nonprofit and private hospitals. In addition, whether or not they receive government evaluations also affects social performance. Hospitals that receive government assessments have lower levels of financial inequity but higher levels of social equity and overall equity compared to hospitals that do not receive government assessments. Publicness factors, such as government certification of hospitals and audits applied to hospitals, do not contribute to improving the social performance of hospitals. In conclusion, this study found that the public ownership factor of hospitals has the greatest influence on the hospital’s social performance. However, we found a significant negative interaction between government ownership and an increase of medical payments. For government owned hospitals, there is a clear tendency that as the number of medical payments increases, equity indicators in our study decrease. The negative interaction was statistically significant in all three equity indicators in the fixed effects models as well as in the random effects models (see Appendix E, Appendix F, Appendix G). The recent trend to emphasize performance management in the public sector involves efficiency pressure that pushes public organizations to support effectiveness rather than equity [31,32]. It appears that government owned public hospitals suffer from efficiency pressure and adopt management strategies for higher efficiency standards. Public hospitals evolve under stresses to promote higher productivity and efficiency.

This study has some limitations for further research. First of all, the measurements of equity outcome of hospitals, in this study, like the ratio of medical aid and veteran patients and the ratio of out-of-pocket payments, also do not fully reflect the level of equity performance of hospitals. For its theoretical development, lots of research on the relationship between publicness and organizational performance, not only in health care but also other discipline areas, needs to be done. Finally, this study does not consider the effect of publicness on the efficiency of hospitals which is also a very important indicator of hospital performance. This topic is another important research area.

Despite these limitations, however, this research contributes to expanding our existing knowledge, adding a new empirical perspective to the hospital performance, especially in terms of equity arising from publicness of the organization in health care. Further research is required to explore how performance management and outsourcing pressure negatively or positively influence performance in health care organizations. There is substantial debate on whether performance management can promote equity and efficiency in public health [33,34]. Future research can explore diverse sectoral characteristics across diverse health organizations [35,36]. The effects of hospital organizational characteristics on performance can differ across public, nonprofit, and profit hospitals. The methods of which hospitals can effectively improve health equity may vary across diverse sectors [37,38]. Competing solutions to a trade-off between efficiency and equity can also vary across government, nonprofit, and for-profit health organizations. In addition, future research is required to explore sectoral diversities to adopt various medical innovation strategies and social externality in health care [39]. Nonprofit hospitals and profit hospitals may be more sensitive to various innovative approaches than public hospitals. In order to improve equity and efficiency, hospital innovation needs to recognize distinctive sector identities and behaviors among health organizations.

## Figures and Tables

**Table 1:** Analytical framework for hospital performance equity.

Dependent Variables		Independent Variables	
**Equity**	**Financial Equity**	Publicness	Ownership (Government, nonprofit, private) Government evaluation Hospital accreditation Accounting audit
	Social Equity		
Overall Equity		Control variables	Number of medical payments Hospital age Number of hospital beds

**Table 2:** Distribution of equity indicators (2008–2012).

		Financial Inequity (*y_1_*)			Social Equity (*y_2_*)			Overall Equity (*y_3_*)		
Year	*N*	Mean	Median	SD	Mean	Median	SD	Mean	Median	SD
2008	311	0.023	0.021	0.010	0.125	0.095	0.090	0.176	0.141	0.113
2009	312	0.019	0.018	0.008	0.114	0.087	0.087	0.161	0.127	0.116
2010	316	0.016	0.015	0.007	0.116	0.087	0.092	0.154	0.122	0.111
2011	319	0.014	0.013	0.006	0.114	0.088	0.086	0.146	0.117	0.100
2012	320	0.014	0.012	0.007	0.105	0.085	0.074	0.134	0.107	0.090
Total	1578	0.017	0.016	0.008	0.115	0.088	0.086	0.154	0.122	0.107

**Table 3:** Distribution of equity indicators across three sectors.

		Financial Inequity (*y_1_*)		Social Equity (*y_2_*)		Overall Equity (*y_3_*)	
	*N*	Mean	Median	Mean	Median	Mean	Median
Government Nonprofit	159	0.01579	0.01507	0.1929	0.1773	0.2634	0.2486
	1090	0.01575	0.01428	0.1101	0.0864	0.1442	0.1149
Private	335	0.02197	0.02009	0.0916	0.0746	0.1348	0.1158

**Table 4:** Distribution of equity indicators by government ownership and evaluation.

			Financial Inequity (*y_1_*)		Social Equity (*y_2_*)		Overall Equity (*y_3_*)	
		*N*	Mean	Median	Mean	Median	Mean	Median
Government Ownership	No	1425	0.0172	0.0157	0.1058	0.0824	0.1420	0.1152
	Yes	159	0.0158	0.0151	0.1929	0.1773	0.2634	0.2486
Govt_Ev	No	1105	0.0187	0.0169	0.0975	0.0820	0.1422	0.1201
	Yes	479	0.0134	0.0130	0.1539	0.1229	0.1818	0.1326

**Table 5:** Distribution of equity indicators (2008–2012) by ownership.

Government Ownership			Financial Inequity (*y_1_*)		Social Equity (*y_2_*)		Overall Equity (*y_3_*)	
	Year	*N*	Mean	Median	Mean	Median	Mean	Median
No	2008	279	0.023	0.021	0.114	0.089	0.161	0.133
	2009	279	0.019	0.018	0.105	0.081	0.149	0.120
	2010	284	0.016	0.015	0.107	0.082	0.142	0.115
	2011	288	0.014	0.013	0.106	0.084	0.135	0.112
	2012	289	0.014	0.012	0.096	0.078	0.123	0.102
	Total	1419	0.017	0.016	0.106	0.082	0.142	0.115
Yes	2008	32	0.020	0.019	0.215	0.195	0.302	0.285
	2009	33	0.017	0.017	0.188	0.174	0.265	0.242
	2010	32	0.014	0.014	0.190	0.176	0.263	0.245
	2011	31	0.014	0.014	0.187	0.171	0.251	0.226
	2012	31	0.013	0.013	0.184	0.174	0.234	0.204
	Total	159	0.016	0.015	0.193	0.177	0.263	0.249

**Table 6:** Distribution of equity indicators (2008–2012) by government evaluation.

Government Evaluation			Financial Inequity (*y_1_*)		Social Equity (*y_2_*)		Overall Equity (*y_3_*)	
	Year	*N*	Mean	Median	Mean	Median	Mean	Median
No	2008	217	0.025	0.023	0.107	0.088	0.163	0.137
	2009	216	0.020	0.019	0.096	0.080	0.147	0.124
	2010	219	0.017	0.016	0.096	0.079	0.141	0.119
	2011	222	0.016	0.014	0.094	0.081	0.135	0.117
	2012	226	0.015	0.013	0.094	0.080	0.126	0.106
	Total	1100	0.019	0.017	0.097	0.082	0.142	0.120
Yes	2008	94	0.018	0.018	0.166	0.136	0.204	0.155
	2009	96	0.015	0.015	0.156	0.128	0.194	0.141
	2010	97	0.013	0.013	0.160	0.126	0.185	0.131
	2011	97	0.011	0.011	0.158	0.125	0.171	0.119
	2012	94	0.011	0.010	0.130	0.098	0.155	0.113
	Total	478	0.013	0.013	0.154	0.123	0.182	0.133

**Table 7:** Fixed effects regression of publicness on financial inequity.

Dependent Variable = y_1_ (Financial Inequity)		Coefficient	SE	t-Value	*p* > |t|
Govt		−0.0393	0.0159	−2.48	0.013
Govt_EV		−0.0022	0.0010	−2.16	0.031
Credit		−0.0004	0.0004	−1.10	0.270
Audit		0.0044	0.0020	2.13	0.033
Private		−0.0236	0.0073	−3.25	0.001
Ln_pay_count		−0.0027	0.0005	−5.89	<0.001
Int_govt		0.0047	0.0018	2.68	0.007
Int_private		0.0028	0.0008	3.43	0.001
Age		0.0000604	0.0000386	1.56	0.118
Bed		0.00000249	0.00000244	1.02	0.308
Year	2009	−0.0035	0.0003	−13.61	<0.001
	2010	−0.0065	0.0003	−24.81	<0.001
	2011	−0.0077	0.0003	−27.54	<0.001
	2012	−0.0083	0.0003	−27.68	<0.001
Intercept		−0.0750	0.0775	−0.97	0.334

Number of observations = 1578; Number of groups = 338. R^2^: Within = 0.565, Between = 0.064, Overall = 0.168; F(14, 1226) = 113.94, Prob. > F < 0.001; Correlation (*u_i_*, Xb) = −0.1401; *σ_u_* = 0.0075; *σ_e_* = 0.0031; ρ(Rho) = 0.85; F-test: All *u_i_* = 0, F(337, 1226), F = 17.81, Prob.> F < 0.001. Hausman Test: Chi square (13) = 51.31, Prob. > Chi square < 0.001.

**Table 8:** Fixed effects regression of publicness on social equity.

Dependent Variable = y_2_ (Social Equity)		Coefficient	SE	t-Value	*p* > |t|
**Govt**		0.5707	0.1579	3.62	<0.001
Govt_Ev		0.1508	0.0099	15.17	<0.001
Credit		−0.0026	0.0036	−0.73	0.463
Audit		−0.0560	0.0203	−2.76	0.006
Private		0.0104	0.0723	0.14	0.886
Ln_pay_count		0.0066	0.0045	1.47	0.141
Int_govt		−0.0607	0.0175	−3.48	0.001
Int_private		−0.0021	0.0080	−0.27	0.791
Age		−0.00004	0.00038	−0.10	0.919
Bed		−0.000042	0.000024	−1.73	0.085
Year	2009	−0.0102	0.0025	−4.02	<0.001
	2010	−0.0066	0.0026	−2.56	0.011
	2011	−0.0065	0.0028	−2.33	0.020
	2012	−0.0130	0.0030	−4.36	<0.001
Intercept		0.1174	0.7711	0.15	0.879

Number of observations = 1578; Number of groups = 338. R2: Within = 0.191; Between = 0.0964; Overall = 0.0955; F(14, 1226) = 20.07, Prob. > F < 0.001 Correlation (*u_i_*, Xb) = −0.443; *σ_u_* = 0.0855; *σ_e_* = 0.0313; ρ(Rho) = 0.8819; F-test: All *u_i_* = 0, F(337, 1226), F = 21.65, Prob. > F < 0.001. Hausman Test: Chi square (13) = 102.43, Prob. > Chi square < 0.001.

**Table 9:** Fixed effects regression of publicness on overall equity.

Dependent Variable = y_3_ (Overall Equity)		Coefficient	SE	t-Value	*p* > |t|
**Govt**		0.8453	0.1855	4.56	<0.001
Govt_Ev		0.0709	0.0117	6.07	<0.001
Credit		−0.0004	0.0042	−0.10	0.920
Audit		−0.0151	0.0239	−0.63	0.526
Private		0.0206	0.0849	0.24	0.808
Ln_pay_count		0.0056	0.0053	1.06	0.290
Int_govt		−0.0943	0.0205	−4.60	<0.001
Int_private		−0.0027	0.0094	−0.29	0.771
Age		−0.0006	0.0005	−1.34	0.182
Bed		0.000028	0.000029	−0.97	0.330
Year	2009	−0.0133	0.0030	−4.47	<0.001
	2010	−0.0178	0.0031	−5.83	<0.001
	2011	−0.0243	0.0033	−7.40	<0.001
	2012	−0.0335	0.0035	−9.57	<0.001
Intercept		1.3112	0.9063	1.45	0.148

Number of observations = 1578; Number of groups = 338. R^2^: Within = 0.1557; Between = 0.0845; Overall = 0.0868; F(14, 1226) = 16.15, Prob. > F < 0.001 Correlation(*u_i_*, Xb) = −0.1589; *σ_u_* = 0.0983; *σ_e_* = 0.0368; ρ(Rho) = 0.8774; F-test: All *u_i_* = 0, F(337, 1226), F = 17.81, Prob. > F < 0.001. Hausman Test: Chi square (13) = 104.15, Prob. > Chi square < 0.001.

## Appendix A

**Table A1:** Definitions of variables and data sources.

Variables	Definitions	Sources
Proportion of out-of-pocket money for the poor (y_1_ = Financial inequity)	The ratio of out-of-pocket payments over medical care expenses for the poor in the Medical Aid program for each hospital	The Health Insurance Review and Assessment Service
Proportion of treatment for the disadvantaged (y_2_ = Social equity)	The ratio of payment statement of medical aid and veteran relief to total payment statements for each hospital	The Health Insurance Review and Assessment Service
The proportion of payment coverage for the disadvantaged (y_3_ = Overall equity)	The ratio of payment of medical aid and veteran relief to total amount of medical payments for each hospital	The Health Insurance Review and Assessment Service
Govt (Dummy variables)	The value of the Govt dummy variable is 1 if the hospital is national, provincial, and city hospitals owned by central and local governments. Otherwise, the value is 0.	The Health Insurance Review and Assessment Service
Govt_EV (Dummy)	The value is 1 if the hospital has legal obligation for government evaluation. Otherwise, the value is 0.	Coding based on related legislation and regulations
Audit (Dummy)	The value is 1 if the hospital has legal obligation for accounting audit. Otherwise, the value is 0.	Coding based on related legislation and regulations
Credit (Dummy)	The value is 1 if the hospital receives accreditation by Korea Institute for Healthcare Accreditation. Otherwise, the value is 0.	Webpage of Korea Institute for Healthcare Accreditation
Ln_Pay_Count	All the medical care statements from health insurance expenses, medical aid expenses, and veterans expenses transformed by natural logarithm	The Health Insurance Review and Assessment Service
Age	Foundation year representing organizational age	Korean Hospital Association (List of Hospitals in Korea)
Bed	The number of hospital beds representing organizational size	The Health Insurance Review and Assessment Service

## Appendix B

**Table A2:** Descriptive statistics.

Variable	Obs	Mean	SD	Min	Max
*y_1_* (Financial inequity)	1578	0.0171	0.0082	0.00005	0.0725
*y_2_* (Social equity)	1578	0.1146	0.0864	0.0047	0.6106
*y_3_* (Overall equity)	1578	0.1542	0.1071	0.0043	0.9942
Govt	1584	0.10	0.30	0	1
Govt_EV	1584	0.30	0.46	0	1
Credit	1584	0.11	0.32	0	1
Audit	1584	0.18	0.38	0	1
Private	1584	0.21	0.41	0	1
Ln_Pay_Count	1578	9.05	1.03	4.14	11.95
Int_govt	1578	0.84	2.53	0	10.44
Int_private	1578	1.81	3.51	0	9.65
Age	1584	1992.24	15.16	1909	2012
Bed	1584	422.30	311.00	100	2680
Year	1584	2010.02	1.41	2008	2012

## Appendix C

**Table A3:** Hospital data coverage for unbalanced panel (2008–2012).

Frequency	Percent	Cumulative	2008	2009	2010	2011	2012
295	87.02	87.02	Yes	Yes	Yes	Yes	Yes
10	2.95	89.97	No	No	Yes	Yes	Yes
7	2.06	92.04	No	No	No	Yes	Yes
6	1.77	93.81	No	Yes	Yes	Yes	Yes
6	1.77	95.58	Yes	Yes	Yes	No	No
5	1.47	97.05	Yes	No	No	No	No
5	1.47	98.53	Yes	Yes	No	No	No
4	1.18	99.71	No	No	No	No	Yes
1	0.29	100	Yes	Yes	Yes	Yes	No
339	100						

Note: ‘Yes’ is an indicator of the availability within a specific period; ‘No’ is not available.

## Appendix D

**Table A4:** Total medical payments of tertiary and general hospitals.

		**National Health Insurance**			
		**Inpatients**		**Outpatients**	
**Year**	**N**	**Mean**	**SD**	**Mean**	**SD**
2008	311	12,603.8	15,094.8	152,844.3	180,090.0
2009	312	13,379.1	16,459.6	178,672.8	209,294.7
2010	316	14,180.1	17,273.0	188,745.7	235,695.9
2011	319	14,623.1	17,471.2	201,214.3	246,827.7
2012	320	15,048.2	17,837.0	276,836.8	336,685.8
		**Medical Care Assistance**			
		**Inpatients**		**Outpatients**	
**Year**	**N**	**Mean**	**SD**	**Mean**	**SD**
2008	311	1774.8	1983.9	14,668.5	13,176.5
2009	312	1670.9	1886.2	16,063.1	15,244.9
2010	316	1719.5	2401.4	17,064.6	17,459.3
2011	319	1806.8	2565.1	18,021.9	18,233.7
2012	320	1463.6	1440.1	21,363.6	17,009.1

Notes: (1) Relying on data from the Health Insurance Review and Assessment Service, authors recalculated these numbers. (2) *N* is the number of tertiary and general hospitals. SD = Standard deviation.

## Appendix E

**Table A5:** Random effects regression of publicness on financial inequity.

		Coefficient	SE	t-Value	*p* > |t|
Govt		−0.024	0.011	−2.070	0.038
Govt_EV		−0.002	0.0008	−2.550	0.011
Credit		0.000	0.00036	−0.730	0.463
Audit		0.000005	0.0011	0.000	0.997
Private		−0.005	0.006	−0.760	0.447
Ln_pay_count		−0.001	0.00036	−3.920	0.000
Int_govt		0.003	0.0013	2.090	0.036
Int_private		0.0009	0.0007	1.350	0.178
Age		0.000039	0.000021	1.810	0.070
Bed		−0.0000016	0.0000015	−1.080	0.282
Year	2009	−0.004	0.00026	−13.670	0.000
	2010	−0.006	0.00026	−24.880	0.000
	2011	−0.008	0.00028	−28.140	0.000
	2012	−0.008	0.00029	−28.520	0.000
Intercept		−0.041	0.043	−0.960	0.337

Number of observations = 1578; Number of groups = 338. R^2^: Within = 0.559, Between = 0.243, Overall = 0.307; Waldo Chi-square(14) = 1646.9, Prob. > Chi-square = 0.0001; Correlation (*u_i_*, X) = 0 assumed; *σ_u_* = 0.006; *σ_e_* = 0.003; ρ(Rho) = 0.798 (fraction of variance due to *u_i_*).

## Appendix F

**Table A6:** Random effects regression of publicness on social equity.

		Coefficient	SE	t-Value	*p* > |t|
Govt		0.2547	0.1179	2.16	0.031
Govt_EV		0.1147	0.0086	13.41	0.000
Credit		−0.0036	0.0036	−0.99	0.324
Audit		−0.0297	0.0110	−2.70	0.007
Private		−0.1220	0.0622	−1.96	0.050
Ln_pay_count		−0.0128	0.0037	−3.41	0.001
Int_govt		−0.0298	0.0136	−2.19	0.029
Int_private		0.0132	0.0072	1.84	0.065
Age		−0.0006	0.0002	−2.51	0.012
Bed		−0.000058	0.000015	−3.81	0.000
Year	2009	−0.0090	0.0026	−3.46	0.001
	2010	−0.0048	0.0026	−1.81	0.070
	2011	−0.0037	0.0028	−1.31	0.190
	2012	−0.0097	0.0030	−3.27	0.001
Intercept		1.3501	0.4517	2.99	0.003

Number of observations = 1578; Number of groups = 338. R^2^: Within = 0.165, Between = 0.204, Overall = 0.199; Waldo Chi-square (14) = 322.3, Prob. > Chi-square = 0.0001; Correlation (*u_i_*, X) = 0 assumed; *σ_u_* = 0.065; *σ_e_* = 0.031; ρ(Rho) = 0.814 (fraction of variance due to *u_i_*).

## Appendix G

**Table A7:** Random effects regression of publicness on overall equity.

		Coefficient	SE	t-Value	*p* > |t|
Govt		0.5537	0.1384	4.00	0.000
Govt_EV		0.0435	0.0100	4.33	0.000
Credit		−0.0021	0.0043	−0.50	0.617
Audit		0.0221	0.0129	1.71	0.088
Private		−0.1604	0.0730	−2.20	0.028
Ln_pay_count		−0.0222	0.0044	−5.06	0.000
Int_govt		−0.0611	0.0160	−3.82	0.000
Int_private		0.0175	0.0084	2.09	0.037
Age		−0.0008	0.0003	−2.95	0.003
Bed		−0.000029	0.000018	−1.64	0.101
Year	2009	−0.0119	0.0031	−3.87	0.000
	2010	−0.0155	0.0031	−5.01	0.000
	2011	−0.0207	0.0033	−6.27	0.000
	2012	−0.0292	0.0035	−8.37	0.000
Intercept		1.9081	0.5295	3.60	0.000

Number of observations = 1578; Number of groups = 338. R^2^: Within = 0.130, Between = 0.299, Overall = 0.287; Waldo Chi-square (14) = 330.9, Prob. > Chi-square = 0.0001; Correlation (*u_i_*, X) = 0 assumed; *σ_u_* = 0.077; *σ_e_* = 0.037; ρ(Rho) = 0.813 (fraction of variance due to *u_i_*).
